# Supplementary material for: GABAA and GABAB Receptors Mediate GABA-Induced Intracellular Ca2+ Signals in Human Brain Microvascular Endothelial Cells
Source: Cells. 2022 Nov 30;11(23):3860. doi: 10.3390/cells11233860 (PMC9739010; doi:10.3390/cells11233860)
Supplement: Supplementary file 1 [file cells-11-03860-s001.zip › cells-2011876-supplementary.pdf]

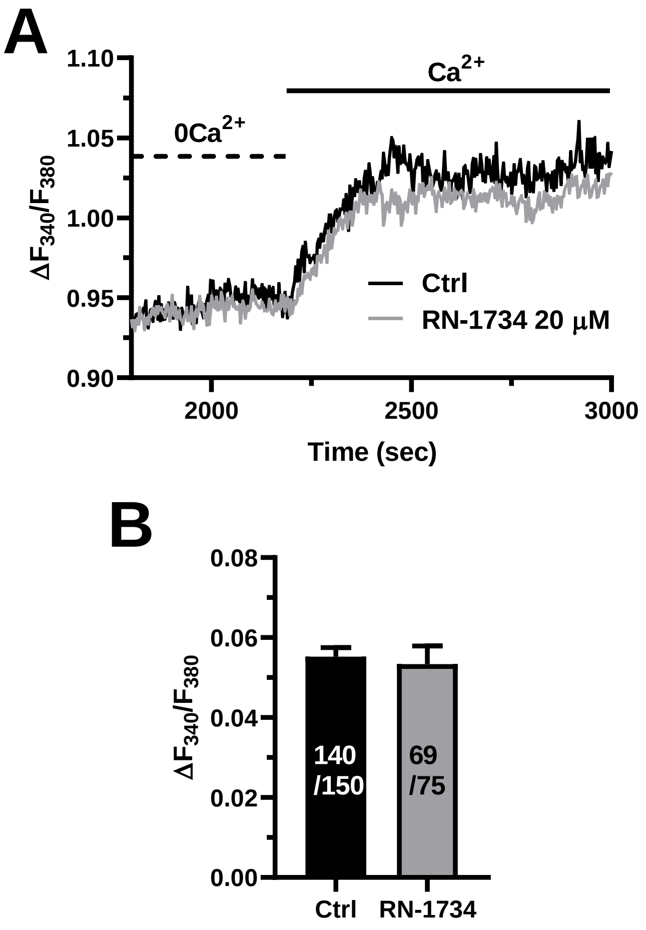


**Supplementary Figure S1. TRPV4 inhibition does not affect GABA-evoked extracellular $Ca^{2+}$ entry in hCMEC/D3 cells.** A, GABA-induced $Ca^{2+}$ influx was not inhibited by blocking TRPV4 channels with RN-1734 (20 µM, 20 min). Intracellular $Ca^{2+}$ release is not shown. B, Mean ± SE of the amplitude of GABA-evoked extracellular $Ca^{2+}$ entry in the absence (Ctrl) and presence of RN-1734.

## Supplementary Table S1. Primers used for real-time

| primer name | sequence 5'-3' | target | amplicon (bp) | Tm | NCBI Reference Sequence | Sequence Name |
|---|---|---|---|---|---|---|
| gabaA-a1_alt_F | TGGAAAGAAGTCAGTGGCCCA | $GABA_A$-α1 | 102 | 61.6 | NM_000806.5 | Homo sapiens gamma-aminobutyric acid type A receptor subunit alpha1 (GABRA1), transcript variant 1, mRNA |
| gabaA-a1_alt_R | GCTCTCACTGTCAGCCTCATGG | | | 62.93 | | |
| gabaA-a2_F | CTGCTTGCCGTTTCAGAGCG | $GABA_A$-α2 | 115 | 62.5 | NM_000807.4 | Homo sapiens gamma-aminobutyric acid type A receptor subunit alpha2 (GABRA2), transcript variant 1, mRNA |
| gabaA-a2_R | ATGTTAGCCAGCACCAACCT | | | 59.59 | | |
| gabaA-a3_F | GCCCGTACAGTCTTTGGTGT | $GABA_A$-α3 | | 60.25 | NM_000808.4 | Homo sapiens gamma-aminobutyric acid type A receptor subunit alpha3 (GABRA3), mRNA |
| gabaA-a3_R | AGACGGCTATGAACCAGTCC | | 118 | 59.18 | | |
| gabaA-a6_F | GACGACCCTAGGAGGGTGAA | $GABA_A$-α6 | | 60.32 | NM_000811.3 | Homo sapiens gamma-aminobutyric acid type A receptor subunit alpha6 (GABRA6), mRNA |
| gabaA-a6_R | AGGGCATTTTCTAGCCACAGA | | 97 | 59.36 | | |
| gabaA-b1_F | TGTGGACAGTACAAAATCGAGAGA | $GABA_A$-β1 | | 59.72 | NM_000812.4 | Homo sapiens gamma-aminobutyric acid type A receptor subunit beta1 (GABRB1), mRNA |
| gabaA-b1_R | TGGGTTCATTGGTGCTGTGT | | 93 | 60.11 | | |
| gabaA-b2_F | TTTACTGGCGTGGCGATGAT | $GABA_A$-β2 | | 60.11 | NM_021911.3 | Homo sapiens gamma-aminobutyric acid type A receptor subunit beta2 (GABRB2), transcript variant 1, mRNA |
| gabaA-b2_R | CCTGGGATAGGAACCTGTGG | | 125 | 59.16 | | |
| gabaA-b3_F | CTGCGCCCAGAGTGTGAAC | $GABA_A$-β3 | | 61.32 | NM_000814.6 | Homo sapiens gamma-aminobutyric acid type A receptor subunit beta3 (GABRB3), transcript variant 1, mRNA |
| gabaA-b3_R | TCGGGTCTTAGGCGAATGTC | | 96 | 59.54 | | |
| gabaA-g1_alt_F | TGCAGAACAGGATCTTGGAGGGA | $GABA_A$-γ1 | 97 | 63.26 | NM_173536.4 | Homo sapiens gamma-aminobutyric acid type A receptor subunit gamma1 (GABRG1), mRNA |
| gabaA-g1_alt_R | ACAGGGCAAAAGCGGTTGGG | | | 63.89 | | |
| gabaA-g2_F | AGCTCAGTCTACTCGACTCCT | $GABA_A$-γ2 | | 52.38 | NM_198904.4 | Homo sapiens gamma-aminobutyric acid type A receptor subunit gamma2 (GABRG2), transcript variant 1, mRNA |
| gabaA-g2_R | CTGGCTAGTGAAGCCAGGGT | | 90 | 60 | | |
| gabaA-g3_F | TTCCCTGTATACTGACTGTGGT | $GABA_A$-γ3 | | 58.21 | NM_033223.5 | Homo sapiens gamma-aminobutyric acid type A receptor subunit gamma3 (GABRG3), transcript variant 1, mRNA |
| gabaA-g3_R | ACCGTGGTGATGCCTAATGC | | 94 | 60.75 | | |
| gabaB1_F | GCCGCTGTGTCCGAATCTGCT | $GABA_B$-1 | 131 | 65.59 | NM_001470.4 | Homo sapiens gamma-aminobutyric acid type B receptor subunit 1 (GABBR1), transcript variant 1, mRNA |
| gabaB1_alt_R | CAGATGGAAGTCGGGGTCACAC | | | 62.87 | | |
| gabaB2_F | TGGAGGCGTCTGTCCATCCGT | $GABA_B$-2 | 93 | 65.62 | NM_005458.8 | Homo sapiens gamma-aminobutyric acid type B receptor subunit 2 (GABBR2), mRNA |
| gabaB2_alt_R | ACAGGCGTGGTTGCAGCAAAA | | | 65.99 | | |
